# News on sickle cell disease: Heme‐driven disordered erythropoiesis

**DOI:** 10.1002/hem3.75

**Published:** 2024-05-17

**Authors:** Francesca Vinchi

**Affiliations:** ^1^ Iron Research Laboratory, Lindsley Kimball Research Institute New York Blood Center New York New York USA; ^2^ Department of Pathology and Laboratory Medicine Weill Cornell Medicine New York New York USA

Sickle cell disease (SCD) is a hemolytic disorder caused by a point mutation in the β‐globin gene leading to the expression of an abnormal hemoglobin (HbS) that has the tendency to polymerize under hypoxic conditions, thus driving red cell sickling. Because of the propensity of sickle red blood cells (RBCs) to break into the circulation and be cleared at a faster rate by reticulo‐endothelial macrophages, hemolysis is a hallmark of SCD.^1^ As a consequence, SCD patients present with elevated circulating heme levels and almost complete exhaustion of the hemoglobin and heme scavengers, haptoglobin, and hemopexin.^2^ The saturation of these scavengers leaves heme in a “free” form, loosely bound to other plasma proteins and thus, more prone to accumulate in cells and tissue and promote cell oxidative damage due to its reactive iron moiety.^2^

Besides hemolysis, disordered erythropoiesis is a feature of hemoglobinopathies, such as β‐thalassemia and SCD. Whereas β‐thalassemia is hallmarked by ineffective erythropoiesis, less is known about alterations in SCD erythropoiesis, which is likely more effective than in β‐thalassemia. A severe misbalance between the expansion of early‐stage erythroid progenitor cells and disrupted differentiation of late‐stage erythroid precursors drive ineffective erythropoiesis in β‐thalassemia.^3^ Although cell death during the Hb synthesis phase of terminal differentiation has been described to contribute to a certain extent to disordered erythropoiesis in SCD,^3^ this mechanism is less pronounced than in β‐thalassemia. Erythroid progenitors in SCD have a better ability to terminally differentiate compared to β‐thalassemia, and peripheral hemolysis of RBCs due to polymerization of deoxygenated sickle hemoglobin is the major cause of anemia in this disease.

**Figure 1:**
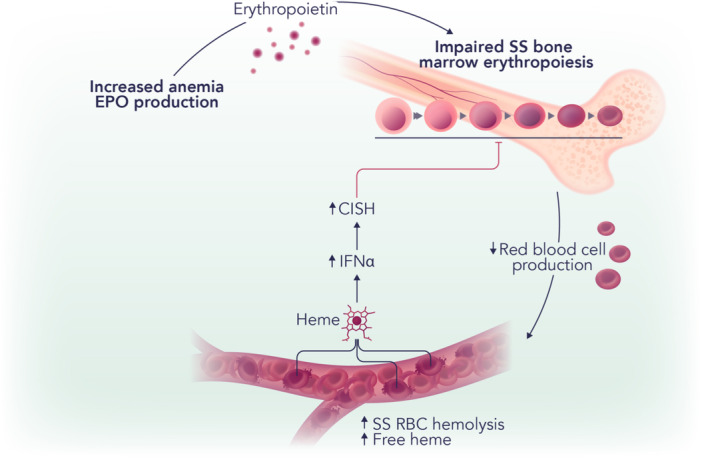
Free heme disrupts erythropoiesis in sickle cell disease (SCD) by driving interferon‐α (IFNα) production. Polymerization of deoxygenated sickle hemoglobin (HbS) in red blood cells (RBCs) leads to erythrocyte sickling in SCD and increases cell fragility leading to extra‐ and intra‐vascular hemolysis and anemia. In this condition, anemia increases hypoxia‐induced erythropoietin (EPO) production by the kidney, which ultimately stimulates erythropoiesis and de novo RBC production in the bone marrow. Upon sickle RBC hemolysis, the release of free heme in the circulation, coupled with the exhaustion of heme scavenging plasma proteins, promotes the production of IFN‐α, which in turn increases the expression of the suppressor of cytokine signaling family member, CISH, in erythroid progenitors. CISH decreases erythropoietin signaling in erythroid cells, resulting in impaired bone marrow erythropoiesis.

The mechanisms of disordered erythropoiesis in SCD, and whether and how hemolysis and free heme contribute to them remained in large part unknown to date. Recently, Xiuli An and group investigated sickle erythropoiesis to better dissect potential alterations in the erythroid activity that could further contribute to anemia, besides the hemolytic process.^4^ Taking advantage of a mouse model of SCD, the authors analyzed bone marrow erythropoiesis and compared it to erythropoiesis in a murine model of β‐thalassemia. Despite similar induction of erythropoietin (EPO) levels, SCD mice exhibited a modest increase in early progenitors and failed to adequately respond to the hormone compared to β‐thalassemia mice, whose erythroblast increase was two‐fold higher than in SCD.^4^ This suggests impaired bone marrow erythropoietic activity, which was confirmed by the observation that SCD erythroid progenitor cells showed decreased erythroid colony‐forming ability and diminished response to EPO in vitro.^4^

Interestingly, hemolysis was found implicated in impaired erythropoiesis in SCD through an indirect mechanism involving interferon‐α (IFNα) (Figure 1). IFNα levels were increased in SCD mice as well as in patients with SCD, where they correlated with circulating heme levels. Indeed, heme injection in mice caused IFNα elevation, proving a direct role for hemolysis in driving IFNα production. Exposure of erythroid precursors to IFNα increased cell expression of an EPO/EPOR‐negative regulator, the suppressor of cytokine signaling family member *Cish*, and decreased their response to EPO.^4^ IFNα receptor deficiency significantly attenuated heme‐induced impairment of bone marrow erythropoiesis. This translated into an improved bone marrow erythropoietic activity as well as EPO‐stimulated erythroid response in SCD mice. Overall, the study by Han et al. identified the heme‐IFNα‐CISH axis as a contributing factor to the disordered erythropoiesis in SCD and provided a mechanistic understanding for the impaired erythropoietic response to EPO, whose levels are already elevated in SCD.^4^

Although gene therapy is upcoming for the treatment of SCD, not all patients are eligible for it. Therefore, most patients rely on classical treatments, including transfusion therapy support, EPO and hydroxyurea, and more recent drugs such as inhibitors of hemoglobin polymerization (e.g., Voxelotor) and endothelial activations (e.g., P‐selectin inhibitor), and antioxidants (e.g., l‐glutamine).^1^, ^5^ The use of EPO has become more widespread in the management of SCD, especially when used in conjunction with hydroxyurea—with the aim to further increase fetal hemoglobin expression. However, the therapeutic doses of EPO necessary to achieve an effect in SCD are considerably higher than those used for other anemic conditions, such as chronic kidney disease.^6^, ^7^, ^8^ The current study offers a plausible explanation of why high EPO doses are required for the treatment of anemia in patients with SCD by describing a novel mechanism of heme‐induced suppression of erythropoiesis via the induction of IFNα expression.^4^

While this study uncovers a new role for heme in suppressing erythropoiesis by inhibiting EPO/EPO receptor signaling via IFNα‐mediated upregulation of *Cish* in erythroid cells, heme might contribute to impaired erythropoiesis in SCD through the production of other proinflammatory cytokines, such as IL‐6, which has a known inhibitory effect on erythropoiesis.^9^

Importantly, the observation that hemolysis suppresses erythropoiesis through IFNα induction likely has implications for other hemolytic anemias or anemia of inflammation, whereby circulating heme or/and IFNα are elevated. Thus, targeting the IFNα pathway and/or heme may offer new treatment options to improve anemia not only in SCD but also in inflammatory and infectious conditions associated with high heme and/or IFNα levels. Understanding the molecular mechanisms underlying IFNα‐mediated suppression of EPO response may lead to the development of novel strategies to improve EPO hyporesponsiveness in SCD as well as in anemia of chronic diseases and inflammation. This work ultimately highlights how, beyond therapeutic strategies that focus on hemolysis, sickle hemoglobin polymerization, and vascular dysfunction, the development of alternative approaches targeting heme‐induced inflammatory response, including IFNα signaling, could provide therapeutic benefit in SCD by improving—in combination with other drugs—disordered erythropoiesis and anemia.

## AUTHOR CONTRIBUTIONS

Francesca Vinchi wrote the HemaTopic and drafted the figure, which was professionally drawn by Somersault18:24 BV.

## CONFLICT OF INTEREST STATEMENT

Dr. Vinchi is a member of the advisory board of Silence Therapeutics and a consultant for RallyBio and Pharmacosmos. None of these relationships is relevant to the current publication.

## FUNDING

This research received no funding.

## Data Availability

Data sharing is not applicable to this article as no data sets were generated or analyzed during the current study.
